# Cost-effectiveness of orbital atherectomy compared to rotational atherectomy in treating patients with severely calcified coronary artery lesions in Japan

**DOI:** 10.1007/s12928-017-0488-3

**Published:** 2017-09-05

**Authors:** Jan B. Pietzsch, Benjamin P. Geisler, Fumiaki Ikeno

**Affiliations:** 1 Wing Tech Inc., Menlo Park, CA USA; 2 Department of Medicine, Massachusetts General Hospital/Harvard Medical School, Boston, MA USA; 3 Division of Cardiovascular Medicine, Stanford University School of Medicine, 300 Pasteur Drive, FALK CVRB CV-007, Stanford, CA 94305 USA

**Keywords:** Vascular calcification, Atherectomy, coronary, Percutaneous coronary intervention, Cost–benefit analysis, Japan

## Abstract

Compared to rotational atherectomy (RA), orbital atherectomy (OA) has been shown to decrease procedure failure and reintervention rates in the treatment of severely calcified coronary artery lesions. Our objective was to explore the cost-effectiveness of OA compared to RA in the Japanese healthcare system. A decision-analytic model calculated reintervention rates and consequent total 1-year costs. Effectiveness inputs were therapy-specific target lesion revascularization (TLR) rates and all-cause mortality, pooled from clinical studies. Index and reintervention costs were determined based on claims data analysis of *n* = 33,628 subjects treated in 2014–2016. We computed incremental cost-effectiveness in Japanese Yen (JPY) per life year (LY) gained based on differences in 1-year cost and projected long-term survival, assuming OA device cost between JPY 350,000 and ​JPY 550,000. OA was found to be associated with improved clinical outcomes (12-month TLR rate 5.0 vs. 15.7%) and projected survival gain (8.34 vs. 8.16 LYs (+0.17), based on 1-year mortality of 5.5 vs. 6.8%). Total 1-year costs were lower for device cost of JPY 430,000 or less, and reached a maximum ICER of JPY 753,445 per LY at the highest assumed device cost, making OA dominant or cost-effective across the tested range, at ICERs substantially below the willingness-to-pay threshold. In conclusion, orbital atherectomy for the treatment of severely calcified coronary artery lesions, compared to rotational atherectomy, is a cost-effective treatment approach in the Japanese healthcare system due to improved clinical performance.

**Electronic supplementary material:**

The online version of this article (doi:10.1007/s12928-017-0488-3) contains supplementary material, which is available to authorized users.

## Introduction

The treatment of severely calcified coronary artery lesions continues to present a clinical and economic challenge. Factors that lead to coronary calcification such as advanced age, diabetes, kidney disease, and smoking are increasing in the US and Japan, and contribute to continued growth of this burden. Compared to less calcified coronary artery lesions, patients with severe calcification have been shown to have worse outcomes [1].

Endovascular treatment of severely calcified coronary lesions is challenging, as these lesions tend to respond poorly to balloon angioplasty, are commonly difficult to completely dilate, and are prone to dissection. Calcium has been found to be associated with stent underexpansion, asymmetric expansion, stent strut malapposition, as well as impaired drug absorption [2, 3]; thus, severely calcified coronary lesions have been excluded from most clinical trials. As a result of this complexity, treatment of severe coronary artery calcification has been shown to lead to longer treatment times, higher resource use, longer hospital stay, and, in consequence, higher treatment costs [1, 4, 5] compared to lesions with little or no calcium.

Atherectomy has emerged as a promising, and sometimes the only viable, treatment option for calcified coronary lesions. Rotational atherectomy (RA) with the Rotablator (Boston Scientific, Natick, MA, USA) is the most widely adopted atherectomy approach for severely calcified coronary lesions to-date in Japan [6, 7], despite having no specific indication for treatment of severe coronary calcium. RA works in a drill-like fashion, producing a 1:1 burr:lumen ratio during treatment of coronary plaque. Orbital atherectomy (OA; Cardiovascular Systems, Inc. St. Paul, MN, USA) is a more recent atherectomy treatment approach with a US Food and Drug Administration indication specific to severe coronary calcium. In contrast to a rotational mechanism of action, OA utilizes centrifugal force allowing for 360° contact of the vessel wall and treatment of variable artery sizes with one device size. Clinical and cost-effectiveness studies of OA in the US have demonstrated reduced procedure complexity and improved patient outcomes [8–10]. OA is commercially marketed only in the US, and is currently awaiting regulatory approval and commercial market entry in Japan.

Our objective was to explore the potential cost-effectiveness of OA compared to RA treatment of severely calcified coronary artery lesions in the Japanese healthcare setting, based on latest cost and clinical data.

## Methods

We developed a decision-analytic model to assess treatment-specific 1-year total cost to Japanese healthcare payers, and to estimate projected life year gain based on trial-observed 12-month mortality data. Based on these data, our primary health-economic outcome measure, the incremental cost-effectiveness ratio (ICER), was computed. The ICER, defined in our study as the incremental direct costs of medical treatment and consequences divided by the incremental health benefits expressed as life years (LYs), is a common metric used in health-economic analyses to assess the value of an intervention [11], and is outlined in Japan’s recently published Guideline for Preparing Cost-Effectiveness Evaluation to the Central Social Insurance Medical Council.

Therapy effectiveness (target lesion revascularization rate, TLR, and all-cause mortality) was determined based on pooled results of clinical studies identified for each strategy through controlled literature review. Where available, studies reporting on Japanese patient populations were used. Cost data were determined from a detailed analysis of claims data from a large Japanese hospital administrative database, and from estimated index procedure device costs and reintervention costs.

### Clinical data

Controlled searches of the published literature were performed in July 2016 to identify relevant clinical studies for inclusion in the analysis. Both prospective and retrospective studies were included. To ensure statistically robust sample sizes, we required sample sizes of *n* = 50 or greater for inclusion. Furthermore, studies had to report 12-month TLRs (or, really, proportions) and/or 12-month all-cause mortality. As unprotected left main lesions were excluded in the present OA studies, we excluded RA studies that report on these lesion types, to ensure comparability.

### Cost data

Procedure costs were derived from a retrospective analysis of cost data from a large hospital administrative database (MDV database, Medical Data Vision, Tokyo, Japan) covering 230 hospitals and 12.94 million patients in Japan. We assumed reimbursement payment amounts to be proxies for true costs and analyzed claims recorded in the period April 2014–March 2016, capturing rotational atherectomy cases (procedure code 150284310), as well as percutaneous coronary intervention (PCI) cases performed with or without stents (procedure codes 150375110 and 150375410, respectively). Both index procedure costs and costs of the first reintervention treatment were determined.

Furthermore, RA device costs were determined based on current Japanese device reimbursement payment amounts for rotational atherectomy devices (Rotablator) and the device-specific guidewire (RotaWire). Total device costs considered the average number of burrs used in the RA procedure, which for the base case was assumed as 1.63 based on Matsuo et al. [12]. OA device costs took into account utilization of 1.08 devices based on weighted pooling of utilization in the ORBIT II and COAST studies [9, 13], and assumed the device-specific guidewire (ViperWire) and one device-specific lubricant (ViperSlide). Whereas OA device costs have not been established in Japan yet, the analysis explored a range of potential reimbursement amounts between JPY 350,000 and JPY 550,000. The lower bound of JPY 350,000 was defined as a reference case reflecting reimbursement for RA, based on JPY 215,000 RA device cost and assumed utilization of 1.63 RA devices per procedure (see Table 1 for details on all additional input assumptions).

**Table 1:** Assumptions for health-economic analysis

Parameter	Value	Source
Rotablator™ index cost	2,504,198	Claims data analysis, 2014–2015
Other PCI index cost (for reference only)	1,584,074	Claims data analysis, 2014–2015
Reintervention cost	1,184,083	Claims data analysis for first TLR, 2014–2015
RA cost, per device	215,000	Current JP reimbursement amount
Number of RA devices used, per procedure	1.63	[12] (combined cohort)
Additional RA cost (RotaWire)	15,400	Current JP reimbursement amount
OA cost, per device	350,000–550,000	Tested range of potential OA device reimbursement in Japan (exploratory)
Number of OA devices used, per procedure	1.08	Weighted average, ORBIT II and COAST studies
Additional OA-related cost (1 ViperWire)	15,400	Assumed same as RotaWire
Additional OA-related cost (1 ViperSlide)	12,400	Assumed cost in absence of finalized reimbursement decision. US list price for ViperSlide is $180
1-year TLR RA	15.7%	Pooled results from 4 JP rotational atherectomy studies [6, 7, 15, 16]
1-year TLR OA**	5.0%	Pooled results from COAST and ORBIT II [9, 13]
1-year All-cause mortality RA	6.8%	Pooled results from 3 JP RA studies [6, 15, 16]
1-year All-cause mortality OA	4.7%	Pooled results from COAST and ORBIT II [9, 13]
Remaining life years at age 74 (1-year post-index)	7.72	Estimate based on Japan lifetables adjusted to account for severely calcified lesion population, using data from [14]—see Appendix for adjustment methodology

All costs expressed in Japanese Yen (JPY)

### Estimation of long-term survival

Differences in long-term survival were based on observed differences in 1-year all-cause mortality between the two treatment strategies, in conjunction with an assessment of the expected remaining life years at the cohort’s age at 1-year follow-up. To adjust age- and gender-specific mortality of the Japanese general population to the cohort of patients with severely calcified coronary artery disease, a relative mortality risk of 2.593 was derived from a study investigating long-term outcome of 25,253 patients with severe coronary artery calcification [14] (see supplementary materials for detailed computations). At age 74 (treatment age of 73 plus 1 year of follow-up), the remaining life expectancy was thus 7.72 years, compared to 13.51 years reported in latest Japanese life tables.

### Model-based calculation of costs and estimated cost-effectiveness

The computational model used the claims data-derived RA index hospitalization costs and subtracted RA device costs to obtain index hospitalization costs excluding devices. The model assumed that these index hospitalization costs would be the same for RA-treated and OA-treated patients. Strategy-specific total index hospitalization costs were thus computed by adding RA device costs and OA device costs, respectively. Furthermore, the model considered estimated reintervention cost through 1 year, on the basis of strategy-specific 12-month TLR data. The cost per reintervention was based on the claims data analysis, and considered the aggregate cost of reintervention across all index procedure types (rotational atherectomy, PCI without stent, and PCI with stent). Total computed 1-year costs were the sum of index procedure and applicable reintervention cost.

Model outcomes were the difference in 1-year cost, and the incremental cost-effectiveness computed as the ratio of cost difference and life year difference between the two strategies. No discounting was applied because of the limited 1-year horizon of the costing analysis.

### Cost-effectiveness computations and sensitivity analyses

The analysis considered a range of potential OA device cost between JPY 350,000 and JPY 550,000, and the assumptions are shown in Table 1. Extensive one-way sensitivity analyses were conducted, exploring the effect of variation in individual model parameters. In the absence of a final reimbursement amount, these sensitivity analyses were performed around a base case scenario defined by an OA device cost that would lead to overall cost neutrality between the two treatment strategies. Parameters tested in sensitivity analysis included cost data, clinical effectiveness data, and mortality data, as specified in Table 1.

## Results

### Clinical data

The conducted search identified a total of four RA studies with combined sample size of 667 patients meeting inclusion criteria [6, 7, 15, 16]. One-year TLR rates in these studies ranged from 9.7 to 21.2%. The weighted average was 15.7%. One-year all-cause mortality was reported in three of the four studies, and ranged from 1.6 to 11.5%, resulting in a weighted average of 6.8%.

For OA, two technologies currently exist, Diamondback 360 Coronary Orbital Atherectomy System Micro Classic Crown (OAS Classic Crown) and Diamondback 360 Coronary Orbital Atherectomy System Micro Crown (OAS Micro Crown). The latter, second-generation technology (OAS Micro Crown) has recently been studied in Japanese and US patients in the COAST study, a prospective single-arm multi-center study of *n* = 100 subjects (ClinicalTrials.gov identifier NCT01092416) [13]. It is currently undergoing regulatory review in Japan and the US, and will be the commercially available OA system in Japan once regulatory approval is granted. The COAST study is the only study reporting on the OAS Micro Crown. The prior generation OAS Classic Crown device has previously been studied in the ORBIT II single-arm, multi-center prospective study (*n* = 443 patients, ClinicalTrials.gov identifier NCT02132611) [9, 10]. As there was no material difference between 1-year MACE and TVR/TLR rates between COAST and ORBIT II (*p* = 0.2228 and *p* = 0.2121, respectively; no overlap of 95% confidence intervals), ORBIT II data were included in our analysis to achieve a higher and more robust sample size (total *n* = 543). One-year TLR rates were 4.7% in ORBIT II and 6.3% in COAST, for weighted average of 5.0%. Twelve-month all-cause mortality was 4.4 and 6.0%, respectively, resulting in a weighted average of 4.7% (see Table 2 for further detail on cohort characteristics).

**Table 2:** Weighted average of clinical cohort characteristics, mean lesion length, and 12-month TLR of included studies for rotational atherectomy and orbital atherectomy

	Rotational atherectomy	Orbital atherectomy
*N*	662	543
Age	72.8	71.3
Male	63.5%	65.8%
Hypertension	82.3%	92.2%
Hyperlipidemia	60.9%	90.4%
Diabetes mellitus	50.0%	36.4%
Smoker	56.7%	67.4%
Previous MI	21.7%	22.2%
Previous CABG	9.0%	11.8%
Previous CVA	12.0%	9.2%
Chronic kidney disease	31.0%	24.0%
On hemodialysis	13.9%	2.4%
Mean lesion length (mm)	28.7	19.3
Twelve-month TLR	15.7%	5.0%

Included data as reported in respective study publication, with missing data points not considered in weighted average

### Costs

Claims data were available for a total of *n* = 33,628 patients undergoing index procedure treatment. *N* = 1373 of these involved RA treatment, with mean patient age of 73.1 years in this subcohort. The mean total index procedure cost was JPY 2,504,198 for RA-treated patients and JPY 1,584,074 for patients treated with other PCI with or without the use of stents. A total of *n* = 7288 first reinterventions were reported in the claims analysis, with average reintervention cost of JPY 1,184,220 (see supplementary materials for further information and distributional information on all index procedure and reintervention costs).

Subtracting estimated RA device cost of JPY 365,850 from the total RA index hospitalization cost yielded a total cost excluding devices of JPY 2,138,348. This amount was used as the basis for OA total cost estimation. Upon adding estimated OA device and accessories cost of JPY 531,080, total index hospitalization cost for OA patients amounted to JPY 2,669,428, a JPY 165,230 increase in cost compared to RA index cost. Based on the identified 12-month TLR rates for OA and RA, 1-year reintervention costs amounted to JPY 59,091 and JPY 185,563 per cohort patient.

### Cost-effectiveness results for tested OA device cost

**Fig. 1:**
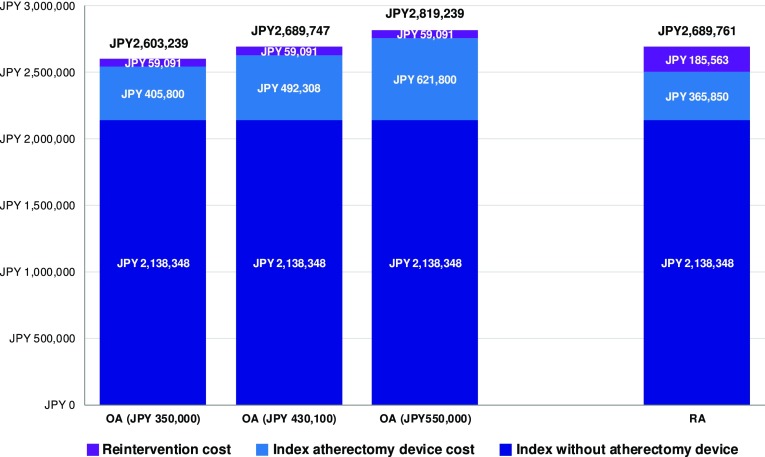
Total one-year costs for RA and OA, by cost type. Columns on the left show results for the three different OA device costs of JPY 350,000 (lower bound), JPY 430,100 (cost-neutral amount), JPY 550,000 (upper bound)

**Fig. 2:**
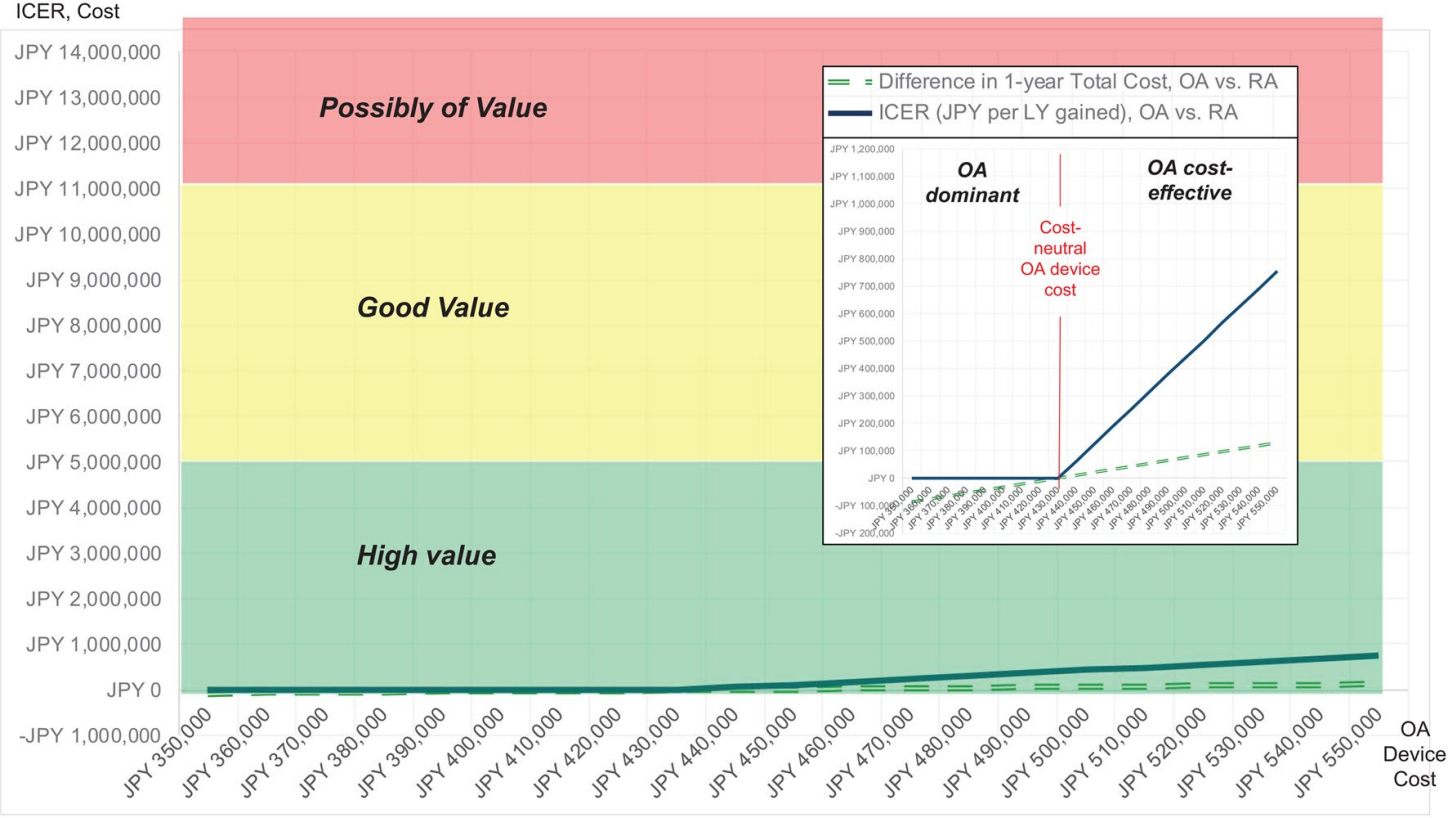
Cost difference (JPY) and ICER (JPY per LY gained) for OA vs. RA therapy, for varying OA device prices between JPY 350,000 and JPY 550,000. Large diagram shows ICER and cost relative to defined value ranges determined by willingness-to-pay thresholds. Small diagram (box) shows same ICER and cost curves in greater resolution

At the lowest tested OA device cost of JPY 350,000, total 1-year costs (index procedure and applicable first reinterventions) were JPY 2,603,239 and JPY 2,689,761 for OA and RA, respectively, resulting in an OA-associated cost savings to healthcare payers of JPY 86,522 (see Fig. 1). Mean projected LYs were 8.34 for OA and 8.16 for RA (+0.17 LYs), resulting in dominance of the OA strategy. OA remained the dominant strategy up to an OA device cost of JPY 430,100, which can hence be considered the cost-neutral device cost. OA device costs higher than this amount led to gradually increasing incremental cost between the two strategies. At the highest tested OA device cost of JPY 550,000, OA was found to be associated with total 1-year cost of JPY 2,819,239, an increase of JPY 129,478 compared to the OA strategy. In conjunction with the OA-associated outcome improvement of 0.17 LY, this resulted in a maximum ICER of JPY 753,445 per LY gained (see Fig. 2).

### Scenario analysis results

Varying the clinical parameter assumptions, assumed device utilization, and device as well as procedure cost assumptions around the cost-neutral base case scenario showed robust findings, with OA being dominant or cost-effective across all tested scenarios, except for an extreme scenario, where RA 1-year mortality was lower than the mortality assumed for the OA base case [see Table 3 for details (as well as extended tables in supplementary materials)].

**Table 3:** Sensitivity analysis results

Description	OA total 1-year cost	RA total 1-year cost	Diff. OA vs. RA 1-year cost	LY OA	LY RA	Diff. OA vs. RA LYs	Cost per LY gained
Base case for scenario analyses (approx. cost-neutral OA device cost of JPY 430,100)	2,689,747	2,689,761	(14)	8.34	8.16	0.17	OA dominant
Variations in TLR, mortality							
OA TLR based on ORBIT II only (4.70%) [9]	2,686,313	2,689,761	(3448)	8.34	8.16	0.17	OA dominant
OA TLR based on COAST only (6.30%) [13]	2,705,260	2,689,761	15,499	8.34	8.16	0.17	90,191
OA all-cause mortality low (4.4%) based on ORBIT II only [9]	2,689,747	2,689,761	(14)	8.36	8.16	0.20	OA dominant
OA all-cause mortality high (6.0%) based on COAST only [13]	2,689,747	2,689,761	(14)	8.23	8.16	0.06	OA dominant
RA TLR low (9.7%), based on [16]	2,689,747	2,619,064	70,683	8.34	8.16	0.17	411,308
RA TLR high (21.2%), based on [15]	2,689,747	2,755,246	(65,500)	8.34	8.16	0.17	OA dominant
RA all-cause mortality low (1.6%), based on [16]	2,689,747	2,689,761	(14)	8.34	8.59	(0.25)	RA cost-effective
RA all-cause mortality high (11.5%), based on [15]	2,689,747	2,689,761	(14)	8.34	7.78	0.56	OA dominant
Variations in device utilization							
Number of OA crowns used low (1.0), per COAST [13]	2,655,339	2,689,761	(34,422)	8.34	8.16	0.17	OA dominant
Number of OA crowns used high (1.1), per ORBIT II [9]	2,698,349	2,689,761	8588	8.34	8.16	0.17	49,976
Number of RA burrs low (1.29) based on [23]	2,762,847	2,689,761	73,086	8.34	8.16	0.17	425,295
Number of RA burrs high (2.175), based on [12] (mean + std. dev.)	2,572,572	2,689,761	(117,189)	8.34	8.16	0.17	OA dominant
Variations in device cost assumptions							
All device costs (OA and RA, including ancillaries) 130% of base case	2,727,684	2,689,761	37,924	8.34	8.16	0.17	220,681
All device costs (OA and RA, including ancillaries) 70% of base case	2,651,809	2,689,761	(37,951)	8.34	8.16	0.17	OA dominant
RA device costs (including ancillaries) 130% of base case	2,579,992	2,689,761	(109,769)	8.34	8.16	0.17	OA dominant
RA device costs (including ancillaries) 70% of base case	2,799,502	2,689,761	109,741	8.34	8.16	0.17	638,594
OA device costs (including ancillaries) 130% of base case	2,837,439	2,689,761	147,679	8.34	8.16	0.17	859,355
OA device costs (including ancillaries) 70% of base case	2,542,054	2,689,761	(147,706)	8.34	8.16	0.17	OA dominant
OA device cost JPY 350,000 (lower bound of tested OA device cost)	2,603,239	2,689,761	(86,522)	8.34	8.16	0.17	OA dominant
OA device cost JPY 550,000 (upper bound of tested OA device cost)	2,819,239	2,689,761	129,478	8.34	8.16	0.17	753,445
Variations in procedure cost assumptions							
RA index procedure cost (also used as basis for OA) high (+30%: 3,255,457)	3,441,006	3,441,020	(14)	8.34	8.16	0.17	OA dominant
RA index procedure cost (also used as basis for OA) low (−30%: 1,752,938)	1,938,488	1,938,501	(14)	8.34	8.16	0.17	OA dominant
Reintervention costs based on reinterventions of RA index	2,705,799	2,740,168	(34,369)	8.34	8.16	0.17	OA dominant
Variations in long-term survival							
Projected remaining life years beyond 1-year post-index low (3.86 years, 50% of base case)	2,689,747	2,689,761	(14)	4.66	4.57	0.09	OA dominant
Projected remaining life years beyond 1-year post-index high (11.58 years, 150% of base case)	2,689,747	2,689,761	(14)	12.02	11.76	0.25	OA dominant

Cost-neutral OA device cost of JPY 430,100 was chosen as base case for this analysis. All cost in JPY. Values in parentheses denote negative amounts. Additional detail provided in supplementary materials

*LY* Life year

## Discussion

Our analysis found current treatment of severely calcified lesions in the Japanese healthcare system to be associated with substantially increased costs compared to treatment of non-calcified lesions. Index procedure costs of patients treated with rotational atherectomy treatment were almost 60% higher than PCI treatment cost in other patients with coronary artery disease. This underscores the substantial economic burden associated with the treatment of severe calcification.

In response to the main objective of our analysis, we explored the potential health-economic profile of orbital atherectomy as a new endovascular treatment option in the Japanese healthcare system. Our findings suggest a promising health-economic profile of OA when compared to RA treatment, with OA projected to provide improved patient outcomes at either cost savings or marginally increased total cost that renders it a high value intervention. These findings result from the substantially (5.0 vs. 15.7%) lower 12-month TLR rate as well as a slightly lower mortality rate (4.7 vs. 6.8%) associated with OA and were robust across a broad spectrum of clinical, utilization, and cost assumptions, with OA showing dominance in scenarios, where OA device costs were lower than JPY 430,100. Across all tested scenarios, the highest incremental cost-effectiveness ratio and, therefore, least favorable result were JPY 753,000, still denoting high value in light of cited Japanese willingness-to-pay thresholds of JPY 5 million to JPY 11 million, as defined in prior publications [17] and the World Health Organization’s recommendation of country-specific willingness-to-pay thresholds of 3 times per-capita GDP.

The results of our study are generally in line with findings of prior studies investigating the cost-effectiveness of OA. Chambers et al. conducted a cost-effectiveness analysis of OA in severely calcified lesions in the US setting, using ORBIT II data to compare OA to other PCI treatment [18]. Similar to the current study—the analysis was found OA to be associated with higher index procedure costs that were more than offset by a reduction in reintervention costs, and with a survival benefit for OA-treated patients. OA was found to be highly cost-effective and likely cost saving. At 2-year follow-up, the attractive value proposition of OA was confirmed in a follow-on study published by the same author group [19]. Shlofmitz and Martinsen performed a retrospective, single-center study of 61 consecutive cases (31 OA vs. 30 non-OA PCI), and likewise found OA to be cost-effective, at a favorable ICER around $15,000 per quality-adjusted life year (QALY) gained. Finally, Chambers and Diage estimated the cost impact of OA treatment to US Medicare payers, based on ORBIT II event rates, and projected savings of $1100 to the healthcare system within the first year [8].

Among the strengths of our analysis is the detailed procedure cost accounting, which was based on a detailed claims data analysis of the Japanese healthcare system. This analysis covered a very recent timeframe (2014–2016), encompassed a comprehensive set of more than 30,000 claims, and provided specific cost information for RA index procedures and for reinterventions. These procedure cost data, in conjunction with a detailed accounting of RA device utilization and current device reimbursement, facilitated a solid assessment of the base cost of interventional treatment of patients presenting with severely calcified coronary artery lesions.

## Limitations

At the same time, our study is subject to several limitations. First, like most model-based analyses, our study makes a number of simplifying assumptions. The cost analysis considered only a 1-year follow-up horizon post-index procedure, and only up to one reintervention—while in reality, some patients might experience more than one reintervention, and also reinterventions at follow-up horizons beyond 1 year. However, most of the clinical data on RA is limited to 1 year of follow-up. Data from those studies reporting longer follow-up—such as ORBIT II [10]—suggest that most reinterventions occur within the first year. Potential consideration of longer follow-up horizons and of more than one reinterventions would likely have made health-economic results for OA more favorable, as it was found to be associated with fewer first reinterventions, and hence likely lower repeat reinterventions. A similar limitation holds for the estimation of life year differences between the two strategies, which was only based on 1-year difference in mortality. Considering 2-year or longer term mortality rates, if available, would have increased the accuracy of projections. Limiting the horizon to 1 year, again, reflected a likely conservative estimate of the outcome benefit associated with OA. Second, while RA data included in the study were—intentionally—limited to results from trials conducted in the Japanese healthcare system, we opted to include OA data from ORBIT II, a large US-based study. However, as has been shown, the outcomes of ORBIT II and of the COAST study, which included US and Japanese patients, did not meaningfully differ. The larger sample size resulting from this approach increases the overall accuracy of the model projections. Furthermore, we explored in sensitivity analyses the effect of using COAST data only. Third, OA is only about to receive regulatory approval in Japan, and reimbursement has not yet been determined for OA. However, in the absence of a fixed reimbursement amount, we explored a broad potential range of device costs, and found OA to be at least cost-effective and potentially dominant across this full range of device costs, underscoring the robustness of the overarching findings. Fourth, device utilization, and specifically the number of burrs used in RA, affects the overarching cost-effectiveness results, and related practice patterns might vary. As the four RA studies included in this analysis did not report absolute numbers of RA burrs used, we relied on data from an earlier study of 200 patients reported by Matsuo et al. [12] to inform device utilization. Their reported number of 1.63 burrs per procedure is in keeping with earlier experience reported in the US [20]. However, there are also a limited number of studies reporting lower utilization as well as higher utilization of RA burrs. Thus, we tested in sensitivity analyses the effect of lower or higher numbers of RA devices used. While reducing the number of RA burrs, as expected, reduced OA-associated cost savings, OA remained cost-effective even for extreme assumptions. Fifth, our analysis results are not discounted, while cost-effectiveness studies reporting longer term projections are typically reported both as discounted and undiscounted values. However, our cost analysis only covered a 1-year timeframe, in which any discounting would have a negligible effect. Furthermore, long-term survival beyond 1 year, aside from difference in 1-year mortality, was assumed to be the same for both strategies, i.e., any differences in outcomes would result from occurrence in the first year. Hence, discounting would have had minimal, if any, effect. Finally, our cost analysis does not take into account potential additional safety benefits and cost reductions that might be associated with the use of OA compared to RA. For example, the smaller size of the OA catheter system facilitates radial as opposed to femoral access. Prior studies have shown reduced access site complications, treatment time, and lower overall treatment cost in patients treated with radial access [21, 22]. Including these aspects would have further increased the health-economic value proposition of OA therapy.

## Conclusions

In summary, our model-based analysis suggests that orbital atherectomy treatment of patients with severely calcified coronary lesions, when compared to rotational atherectomy treatment, is associated with a lower target lesion revascularization rate and represents a cost-effective treatment strategy. Future confirmatory analyses are warranted.

## Electronic supplementary material

Below is the link to the electronic supplementary material.

Supplementary material 1 (DOCX 210 kb)
