# Correction to: Cost-effectiveness of orbital atherectomy compared to rotational atherectomy in treating patients with severely calcified coronary artery lesions in Japan

**DOI:** 10.1007/s12928-017-0492-7

**Published:** 2017-10-27

**Authors:** Jan B. Pietzsch, Benjamin P. Geisler, Fumiaki Ikeno

**Affiliations:** 1 Wing Tech Inc., Menlo Park, CA USA; 2 Department of Medicine, Massachusetts General Hospital/Harvard Medical School, Boston, MA USA; 3 Division of Cardiovascular Medicine, Stanford University School of Medicine, 300 Pasteur Drive, FALK CVRB CV-007, Stanford, CA 94305 USA

## Correction to: Cardiovasc Interv and Ther DOI 10.1007/s12928-017-0488-3

In the original publication of this article, in Abstract the 1-year mortality of OAS should be stated as 4.7 and not 5.5%.

