# Supplementary material for: Cost-effectiveness of orbital atherectomy compared to rotational atherectomy in treating patients with severely calcified coronary artery lesions in Japan
Source: Cardiovasc Interv Ther. 2017 Sep 5;33(4):328–36. doi: 10.1007/s12928-017-0488-3 (PMC6153894; doi:10.1007/s12928-017-0488-3)
Supplement: Supplementary file 1 — Supplementary material 1 (DOCX 210 kb) [file 12928_2017_488_MOESM1_ESM.docx]

**Cost-Effectiveness of Orbital Atherectomy compared to Rotational Atherectomy in Treating Patients with Severely Calcified Coronary Artery Lesions in Japan**

Fumiaki Ikeno, MD; Benjamin P. Geisler, MD, MPH; Jan B. Pietzsch, PhD

**ELECTRONIC SUPPLEMENTARY MATERIALS**

1. **Systematic Literature Search:**

On October 25, 2016, the following search was conducted using PubMed to identify published articles (last 10 years) pertaining to Rotablator/Rotational Atherectomy studies. The initial search included trial cohort sizes greater than 25 patients. This search yielded a total of N=22 studies.

Per the requirements of the current health-economic study, we required studies to report on at least 50 patients, to not exclusively report on hemodialysis patients, and to report 12-month TLR data. This led to the final four identified studies[1-4]. See detail below for search strategy and PRISMA diagram.

**Search Details**

| **User query:** |
| --- |
| (rotablator OR rotablation OR rotational atherectomy) AND ("last 10 years"[PDat]) |
| **Query Translation:** |
| (rotablator[All Fields] OR ("atherectomy, coronary"[MeSH Terms] OR ("atherectomy"[All Fields] AND "coronary"[All Fields]) OR "coronary atherectomy"[All Fields] OR "rotablation"[All Fields]) OR ("atherectomy, coronary"[MeSH Terms] OR ("atherectomy"[All Fields] AND "coronary"[All Fields]) OR "coronary atherectomy"[All Fields] OR ("rotational"[All Fields] AND "atherectomy"[All Fields]) OR "rotational atherectomy"[All Fields])) AND ("2006/10/29"[PDat] : "2016/10/25"[PDat]) |
| **Result:** |
| [588](https://www.ncbi.nlm.nih.gov/pubmed?cmd=HistorySearch&querykey=1) |
| **Translations:** |
| \| rotablation \| "atherectomy, coronary"[MeSH Terms] OR ("atherectomy"[All Fields] AND "coronary"[All Fields]) OR "coronary atherectomy"[All Fields] OR "rotablation"[All Fields] \| \| --- \| --- \| \| rotational atherectomy \| "atherectomy, coronary"[MeSH Terms] OR ("atherectomy"[All Fields] AND "coronary"[All Fields]) OR "coronary atherectomy"[All Fields] OR ("rotational"[All Fields] AND "atherectomy"[All Fields]) OR "rotational atherectomy"[All Fields] \| |

**Literature Analysis Flow Chart**

Exclude animal studies

Exclude studies of < 25 patients

Include only studies of Japanese patients

Literature retrieved for more detailed assessment: 30 articles

Potentially relevant literature identified through the search: 588 articles

Exclude articles based on title/abstract: articles excluded (558)

Literature meeting inclusion criteria per economic study requirements: 4 articles

Exclude articles not reporting 12-month TLR data and studies reporting less than 50 patients (per economic analysis requirements): (18)

Relevant literature:

22 articles

Literature excluded based on review of article (8):

See Table 1 for rationale

**Table A-1**. Articles Identified from PubMed Literature Search (25Oct2016) Obtained for Full Article Review

| **Citation** | **Inclusion/Exclusion** | | **Rationale if Publication Not Used** |
| --- | --- | --- | --- |
|  | **Excluded based on review of article** | **Included in Japan Rota summary** |  |
| 1. Mori T, Sakakura K, Wada H, Taniguchi Y, Yamamoto K, Adachi Y, Funayama H, Momomura SI, Fujita H. [Comparison of mid-term clinical outcomes between on-label and off-label use of rotational atherectomy.](https://www.ncbi.nlm.nih.gov/pubmed/27709324) Heart Vessels. 2016 Oct 5. |  | X |  |
| 1. Aminian A, Iglesias JF, Van Mieghem C, Zuffi A, Ferrara A, Manih R, Dolatabadi D, Lalmand J, Saito S. [First prospective multicenter experience with the 7 French Glidesheath slender for complex transradial coronary interventions.](https://www.ncbi.nlm.nih.gov/pubmed/27567021) Catheter Cardiovasc Interv. 2016 Aug 27. doi: 10.1002/ccd.26773. | X |  | Study not conducted in Japan; results not specific to Rotablator |
| 1. Sakakura K, Funayama H, Taniguchi Y, Tsurumaki Y, Yamamoto K, Matsumoto M, Wada H, Momomura SI, Fujita H. [The incidence of slow flow after rotational atherectomy of calcified coronary arteries: A randomized study of low speed versus high speed.](https://www.ncbi.nlm.nih.gov/pubmed/27453426) Catheter Cardiovasc Interv. 2016 Jul 25. doi: 10.1002/ccd.26698. |  | X |  |
| 1. Maejima N, Hibi K, Saka K, Akiyama E, Konishi M, Endo M, Iwahashi N, Tsukahara K, Kosuge M, Ebina T, Umemura S, Kimura K. [Relationship Between Thickness of Calcium on Optical Coherence Tomography and Crack Formation After Balloon Dilatation in Calcified Plaque Requiring Rotational Atherectomy.](https://www.ncbi.nlm.nih.gov/pubmed/27087360) Circ J. 2016 May 25;80(6):1413-9. doi: 10.1253/circj.CJ-15-1059. | X |  | Imaging study only |
| 1. Isogai T, Yasunaga H, Matsui H, Tanaka H, Fushimi K. [Relationship between hospital volume and major cardiac complications of rotational atherectomy: A nationwide retrospective cohort study in Japan.](https://www.ncbi.nlm.nih.gov/pubmed/26271446) J Cardiol. 2016 May;67(5):442-8. doi: 10.1016/j.jjcc.2015.07.008. |  | X |  |
| 1. Akutsu Y, Hamazaki Y, Sekimoto T, Kaneko K, Kodama Y, Li HL, Suyama J, Gokan T, Sakai K, Kosaki R, Yokota H, Tsujita H, Tsukamoto S, Sakurai M, Sambe T, Oguchi K, Uchida N, Kobayashi S, Aoki A, Kobayashi Y. [Dataset of calcified plaque condition in the stenotic coronary artery lesion obtained using multidetector computed tomography to indicate the addition of rotational atherectomy during percutaneous coronary intervention.](https://www.ncbi.nlm.nih.gov/pubmed/26977441) Data Brief. 2016 Feb 27;7:376-80. doi: 10.1016/j.dib.2016.02.052. | X |  | Imaging study only |
| 1. Tamura H, Miyauchi K, Dohi T, Tsuboi S, Ogita M, Kasai T, Okai I, Katoh Y, Miyazaki T, Naito R, Konishi H, Yokoyama K, Okazaki S, Isoda K, Kurata T, Daida H. [Comparison of Clinical and Angiographic Outcomes After Bare Metal Stents and Drug-Eluting Stents Following Rotational Atherectomy.](https://www.ncbi.nlm.nih.gov/pubmed/26973257) Int Heart J. 2016;57(2):150-7. doi: 10.1536/ihj.15-222. |  | X |  |
| 1. Sekimoto T, Akutsu Y, Hamazaki Y, Sakai K, Kosaki R, Yokota H, Tsujita H, Tsukamoto S, Kaneko K, Sakurai M, Kodama Y, Li HL, Sambe T, Oguchi K, Uchida N, Kobayashi S, Aoki A, Gokan T, Kobayashi Y. [Regional calcified plaque score evaluated by multidetector computed tomography for predicting the addition of rotational atherectomy during percutaneous coronary intervention.](https://www.ncbi.nlm.nih.gov/pubmed/26811266) J Cardiovasc Comput Tomogr. 2016 May-Jun;10(3):221-8. doi: 10.1016/j.jcct.2016.01.004. | X |  | Imaging study only; < 25 patients |
| 1. Dai Y, Takagi A, Konishi H, Miyazaki T, Masuda H, Shimada K, Miyauchi K, Daida H. [Long-term outcomes of rotational atherectomy in coronary bifurcation lesions.](https://www.ncbi.nlm.nih.gov/pubmed/26668644) Exp Ther Med. 2015 Dec;10(6):2375-2383. |  | X |  |
| 1. Kozuma K, Otsuka M, Ikari Y, Uehara Y, Yokoi H, Sano K, Tanabe K, Hibi K, Yamane M, Ishiwata S, Ohta H, Yamauchi Y, Suematsu N, Nakayama M, Inoue N, Kyono H, Suzuki N, Isshiki T. [Clinical and angiographic outcomes of paclitaxel-eluting coronary stent implantation in hemodialysis patients: A prospective multicenter registry: The OUCH-TL study (outcome in hemodialysis of TAXUS Liberte).](https://www.ncbi.nlm.nih.gov/pubmed/25847090) J Cardiol. 2015 Dec;66(6):502-8. doi: 10.1016/j.jjcc.2015.03.008. | X |  | Results not specific to Rotablator |
| 1. Otsuka M, Shiode N, Masaoka Y, Okimoto T, Tamekiyo H, Kawase T, Yamane K, Kagawa Y, Hironobe N, Higashihara T, Fujii Y, Hayashi Y. [Comparison of everolimus- and paclitaxel-eluting stents in dialysis patients.](https://www.ncbi.nlm.nih.gov/pubmed/25953112) Cardiovasc Revasc Med. 2015 Jun;16(4):208-12. doi: 10.1016/j.carrev.2015.04.005. | X |  | Results not specific to Rotablator |
| 1. Tsutsumi J, Ishikawa T, Nakano Y, Yoshimura M, Mutoh M. [Long-term clinical and angiographic outcomes after sirolimus- and paclitaxel-eluting stent placement following rotablation for severely calcified lesions: a retrospective nonrandomized study.](https://www.ncbi.nlm.nih.gov/pubmed/25069959) Cardiovasc Interv Ther. 2015 Jan;30(1):29-37. doi: 10.1007/s12928-014-0283-3. |  | X |  |
| 1. Jinnouchi H, Kuramitsu S, Shinozaki T, Kobayashi Y, Hiromasa T, Morinaga T, Mazaki T, Sakakura K, Soga Y, Hyodo M, Shirai S, Ando K. [Two-Year Clinical Outcomes of Newer-Generation Drug-Eluting Stent Implantation Following Rotational Atherectomy for Heavily Calcified Lesions.](https://www.ncbi.nlm.nih.gov/pubmed/26073607) Circ J. 2015;79(9):1938-43. doi: 10.1253/circj.CJ-15-0233. |  | X |  |
| 1. Lee Y, Tanaka A, Mori N, Yoshimura T, Nakamura D, Taniike M, Makino N, Egami Y, Shutta R, Tanouchi J, Nishino M. [Thin-strut drug-eluting stents are more favorable for severe calcified lesions after rotational atherectomy than thick-strut drug-eluting stents.](https://www.ncbi.nlm.nih.gov/pubmed/24486659) J Invasive Cardiol. 2014 Feb;26(2):41-5. |  | X |  |
| 1. Yabushita H, Takagi K, Tahara S, Fujino Y, Warisawa T, Kawamoto H, Watanabe Y, Mitomo S, Karube K, Matsumoto T, Sato T, Naganuma T, Kobayashi T, Ishiguro H, Fukino K, Kurita N, Nakamura S, Hozawa K, Nakamura S. [Impact of rotational atherectomy on heavily calcified, unprotected left main disease.](https://www.ncbi.nlm.nih.gov/pubmed/24920410) Circ J. 2014;78(8):1867-72. |  | X |  |
| 1. Nishida K, Kimura T, Kawai K, Miyano I, Nakaoka Y, Yamamoto S, Kaname N, Seki S, Kubokawa S, Fukatani M, Hamashige N, Morimoto T, Mitsudo K; j-Cypher Registry Investigators. [Comparison of outcomes using the sirolimus-eluting stent in calcified versus non-calcified native coronary lesions in patients on- versus not on-chronic hemodialysis (from the j-Cypher registry).](https://www.ncbi.nlm.nih.gov/pubmed/23707041) Am J Cardiol. 2013 Sep 1;112(5):647-55. doi: 10.1016/j.amjcard.2013.04.043. |  | X |  |
| 1. Tsujita H, Hamazaki Y, Nishikura T, Yokota H, Kondo S, Hosokawa S, Tsukamoto S, Mutou M, Sakurai M, Nishimura H, Kobayashi Y. [Sirolimus-eluting stents versus paclitaxel-eluting stents for coronary intervention in patients with renal failure on hemodialysis.](https://www.ncbi.nlm.nih.gov/pubmed/23054964) Cardiovasc Interv Ther. 2013 Jan;28(1):9-15. doi: 10.1007/s12928-012-0103-6. |  | X |  |
| 1. Sakakura K, Ako J, Wada H, Naito R, Funayama H, Arao K, Kubo N, Momomura S. [Comparison of frequency of complications with on-label versus off-label use of rotational atherectomy.](https://www.ncbi.nlm.nih.gov/pubmed/22579342) Am J Cardiol. 2012 Aug 15;110(4):498-501. doi: 10.1016/j.amjcard.2012.04.021. |  | X |  |
| 1. Sakakura K, Ako J, Wada H, Naito R, Arao K, Funayama H, Kubo N, Momomura S. [Beta-blocker use is not associated with slow flow during rotational atherectomy.](https://www.ncbi.nlm.nih.gov/pubmed/22865307) J Invasive Cardiol. 2012 Aug;24(8):379-84. |  | X |  |
| 1. Furuichi S, Tobaru T, Asano R, Watanabe Y, Takamisawa I, Seki A, Sumiyoshi T, Tomoike H. [Rotational atherectomy followed by cutting-balloon plaque modification for drug-eluting stent implantation in calcified coronary lesions.](https://www.ncbi.nlm.nih.gov/pubmed/22562910) J Invasive Cardiol. 2012 May;24(5):191-5. |  | X |  |
| 1. Naito R, Sakakura K, Wada H, Funayama H, Sugawara Y, Kubo N, Ako J, Momomura S. [Comparison of long-term clinical outcomes between sirolimus-eluting stents and paclitaxel-eluting stents following rotational atherectomy.](https://www.ncbi.nlm.nih.gov/pubmed/22790681) Int Heart J. 2012;53(3):149-53. |  | X |  |
| 1. Kyono H, Kozuma K, Shiratori Y, Maeno Y, Iino R, Takada K, Ishikawa S, Konno K, Yamamoto H, Suzuki N, Miyazawa A, Yamakawa T, Yokoyama N, Isshiki T. [Angiographic and clinical outcomes of 100 consecutive severe calcified lesions requiring rotational atherectomy prior to sirolimus-eluting stent implantation in hemodialysis and non-hemodialysis patients.](https://www.ncbi.nlm.nih.gov/pubmed/24122529) Cardiovasc Interv Ther. 2011 May;26(2):98-103. doi: 10.1007/s12928-010-0042-z. |  | X |  |
| 1. Kubota T, Ishikawa T, Nakano Y, Endoh A, Suzuki T, Sakamoto H, Hasuda T, Imai K, Yoshimura M, Mutoh M. [Retrospective comparison of clinical and angiographic outcomes after sirolimus-eluting and bare-metal stent implantation in 312 consecutive, nonrandomized severely calcified lesions using a rotablator.](https://www.ncbi.nlm.nih.gov/pubmed/21483162) Int Heart J. 2011;52(2):65-71. |  | X |  |
| 1. Rathore S, Matsuo H, Terashima M, Kinoshita Y, Kimura M, Tsuchikane E, Nasu K, Ehara M, Asakura Y, Katoh O, Suzuki T. [Rotational atherectomy for fibro-calcific coronary artery disease in drug eluting stent era: procedural outcomes and angiographic follow-up results.](https://www.ncbi.nlm.nih.gov/pubmed/20432398) Catheter Cardiovasc Interv. 2010 May 1;75(6):919-27. doi: 10.1002/ccd.22437. |  | X |  |
| 1. Fujimoto H, Ishiwata S, Yamaguchi T, Ohno M. [Usefulness of rotational atherectomy for the implantation of drug-eluting stents in the calcified lesions of hemodialysis patients.](https://www.ncbi.nlm.nih.gov/pubmed/20206077) J Cardiol. 2010 Mar;55(2):232-7. doi: 10.1016/j.jjcc.2009.11.003. |  | X |  |
| 1. Tamekiyo H, Hayashi Y, Toyofuku M, Ueda H, Sakuma T, Okimoto T, Otsuka M, Imazu M, Kihara Y. [Clinical outcomes of sirolimus-eluting stenting after rotational atherectomy.](https://www.ncbi.nlm.nih.gov/pubmed/19749479) Circ J. 2009 Nov;73(11):2042-9. |  | X |  |
| 1. Furuichi S, Sangiorgi GM, Godino C, Airoldi F, Montorfano M, Chieffo A, Michev I, Carlino M, Colombo A. [Rotational atherectomy followed by drug-eluting stent implantation in calcified coronary lesions.](https://www.ncbi.nlm.nih.gov/pubmed/19736163) EuroIntervention. 2009 Aug;5(3):370-4. | X |  | Study not conducted in Japan |
| 1. Kawaguchi R, Tsurugaya H, Hoshizaki H, Toyama T, Oshima S, Taniguchi K. [Impact of lesion calcification on clinical and angiographic outcome after sirolimus-eluting stent implantation in real-world patients.](https://www.ncbi.nlm.nih.gov/pubmed/18206630) Cardiovasc Revasc Med. 2008 Jan-Mar;9(1):2-8. doi: 10.1016/j.carrev.2007.07.004. | X |  | Results not specific to Rotablator |
| 1. Matsuo H, Watanabe S, Watanabe T, Warita S, Kojima T, Hirose T, Iwama M, Ono K, Takahashi H, Segawa T, Minatoguchi S, Fujiwara H. [Prevention of no-reflow/slow-flow phenomenon during rotational atherectomy--a prospective randomized study comparing intracoronary continuous infusion of verapamil and nicorandil.](https://www.ncbi.nlm.nih.gov/pubmed/17967610) Am Heart J. 2007 Nov;154(5):994.e1-6. |  | X |  |
| 1. Iwasaki K, Samukawa M, Furukawa H. [Comparison of the effects of nicorandil versus verapamil on the incidence of slow flow/no reflow during rotational atherectomy.](https://www.ncbi.nlm.nih.gov/pubmed/17134628) Am J Cardiol. 2006 Nov 15;98(10):1354-6. |  | X |  |

1. **Patient and Lesion Characteristics at Baseline:**

The following tables describe baseline characteristics of patients, lesions, and their treatment for the rotational and the orbital atherectomy studies

**Table A-2**. Baseline Patient and Lesion Characteristics of the Included Rotational Atherectomy Studies

| **First author/year** | | **Jinnouchi 2015** | **Nishida 2013** | **Kyono 2011** | **Tamekiyo 2009** |
| --- | --- | --- | --- | --- | --- |
| **Arm (if applicable)** | |  | **RA, non-HD arm** | **non-HD arm** | **RA-SES** |
| **N** | | 252 | 268 | 63 | 79 |
| **Patient baseline  characteristics** | Age | 73.2 | 73 | 72.7 | 70.6 |
|  | Gender (% male) | 61.5% | 63.0% | 70.6% | 65.8% |
|  | Hypertension | 84.1% | 81.0% | 88.2% | 76.0% |
|  | Hyperlipidemia | 60.7% |  | 61.8% |  |
|  | Diabetes mellitus | 50.8% | 46.0% | 63.2% | 50.6% |
|  | Current smoker | 10.7% | 8.2% |  |  |
|  | Ever smoker |  |  | 56.7% |  |
|  | Family history of CAD | 4.8% |  | 23.5% |  |
|  | Chronic kidney disease | 31.0% |  |  |  |
|  | On hemodialysis | 23.0% | 0.0% | 0.0% | 43.0% |
| **Proportion of MACCEs and previous interventions** | Previous MI | 17.9% | 24.0% | 39.8% | 11.4% |
|  | Previous PCI | 31.7% | 39.0% |  | 39.2% |
|  | Previous CABG | 7.5% | 12.0% | 5.9% | 6.3% |
|  | Previous CVA | 11.9% | 12.0% |  |  |
|  | Previous PAD | 28.1% | 15.0% |  |  |
| **Lesion  characteristics** | Calcification level | severe | severe | severe | 74.2% |
|  | ACC/AHA Classification B2/C | 83.1% |  | 100.0% | 98. 0% |
|  | Chronic total occlusion | 8.1% | 12.0% | 4.20% |  |
|  | Bifurcation lesion | 24.5% | 21.0% |  |  |
|  | Minimum lumen diameter, pre (mm) | 0.58±0.30 |  | 0.89±0.33 | 0.76±0.44 |
|  | Minimum lumen diameter, post (mm) | 2.22±0.40 |  | 2.22±0.40 | 2.67±0.56 |
|  | Reference diameter (mm) | 2.47±0.49 |  | 2.20±0.45 | 2.80±0.62 |
|  | Mean Lesion length (mm) | 31.9±15.6 |  | 23.2±16.8 | 22.9±15.9 |

ACC/AHA: American College of Cardiology/American Heart Association; CABG: coronary artery bypass graft; CAD: coronary artery disease; CVA: cerebrovascular accident; MACCE: major adverse cardio-/cerebrovascular events; MI: myocardial infarction; PAD: peripheral artery disease; PCI: percutaneous coronary intervention

**Table A-3**. Reported Treatment(s) of the Lesions in the Included Orbital Atherectomy Studies

| **First author/year** | | | **Jinnouchi 2015** | **Nishida 2013** | **Kyono 2011** | **Tamekiyo 2009** |
| --- | --- | --- | --- | --- | --- | --- |
| **Arm (if applicable)** | | |  | **RA, non-HD arm** | **non-HD arm** | **RA-SES** |
| **Treatment** | Type | | RA + DES | RA + SES | RA + SES | RA + SES |
|  | Number of patients | | 273 | 268 | 63 | 79 |
|  | Balloon used | | NA | NA | 77.8% | NA |
|  | Stent/Balloon Max Pressure, atm | | 13.6±4.3 | 18.4±3.3 | 19.2±2.6 | 19.5±3.59 |
| **Stent** | Type of stent used | | DES | SES | SES | SES |
|  | Stent length, mm | | 32 [23–52] | 32.4±18.5 | 50.6±21.7 | 36.5±17.2 |
|  | Number of stents used per lesion | | 1 | 1.4±0.7 | 2.03±0.70 | 1.65±0.72 |
|  | Number of stents per patient | | NA | 2.5±1.7 | NA | NA |
|  | Stent diameter, mm | | 2.75 [2.5–3.0] | NA | 2.89±0.28 | 2.77±0.31 |
| **Device, Burrs, and Drilling** | RA device | | Rotablator | NA | Rotablator | Rotablator |
|  | Number of burrs | | 1 (57.1%);  2 (42.1%) | NA | NA | NA |
|  | Burr/artery ratio | | NA | NA | 0.66±0.13 | 0.66±0.13 |
|  | Size of burr | | 1.5 [1.5–1.75] | NA | 1.69±0.20 | 1.78±0.27 |
|  | Speed, rpm | | 180k - 210k | NA | NA | 190k - 210k |
| **Lesion** | Calcification level | | Severe | Moderate to Severe | Severe | Severe (74.2%) |
|  | Number of Vessels treated, per patient | |  | 1.4±0.6 | NA | 1.90±0.73 |
|  | Number of lesions treated, per patient | |  | 1.6±0.8 | 1.14 | NA |
|  | Culprit lesion | |  |  |  |  |
|  |  | Right coronary artery | 27.8% | 30.0% | 18.1% | NA |
|  |  | Left anterior descending artery | 55.3% | 48.0% | 61.1% | NA |
|  |  | Left circumflex artery | 11.7% | 17.0% | 6.9% | NA |
|  |  | Left main trunk | 5.1% | 4.6% | 13.9% | NA |
|  | ACC/AHA lesion classification | |  |  |  |  |
|  |  | A | 0.0% | NA | NA | NA |
|  |  | B1 | 16.9% | NA | NA | NA |
|  |  | B2 | 39.9% | NA | NA | NA |
|  |  | C | 43.2% | NA | NA | NA |
|  |  | B2&C | 83.1% | NA | 100.0% | 98.0% |
|  | Chronic total occlusion | | 8.1% | 12.0% | 4.2% | NA |
|  | Bifurcation lesion | | 24.5% | 21.0% | NA | NA |
|  | Tortuosity | | 5.1% | NA | NA | NA |
|  | Reference diameter, mm | | 2.47±0.49 | NA | 2.20±0.45 | 2.80±0.62 |
|  | Diameter stenosis rate, % | | 76.4±11.8 | NA | 60.3±14.0 | NA |
|  | Minimum lumen diameter, mm | | 0.58±0.30 | NA | 0.89±0.33 | 0.76±0.44 |
|  | Lesion length, mm | | 31.9±15.6 | NA | 23.2±16.8 | 22.9±15.9 |
|  | Procedural success rate | | 98.90% | NA | 93.10% | NA |
| **Post procedure** | Reference diameter, mm | | 2.68±0.43 | NA | *2.63±0.74 | NA |
|  | Diameter stenosis rate, % | | 17.1±7.0 | NA | *16.2±8.75 | NA |
|  | Minimum lumen diameter, mm | | 2.22±0.40 | NA | *2.19±0.43 | 2.67±0.56 |
|  | Acute gain, mm | | 1.64±0.42 | NA | 0.96±0.52 | 1.91±0.50 |
| **Follow-up** | Time (months) | | 24 | 60 | 12 | 12 |
|  | Reference diameter, mm | | 2.54±0.47 | NA | *2.59±0.54 | NA |
|  | Diameter stenosis rate, % | | 31.5±22.8 | NA | *27.4±16.9 | NA |
|  | Minimum diameter, mm | | 1.75±0.68 | NA | *1.88±0.60 | 1.90±0.11 |
|  | Late lumen loss, mm | | 0.46±0.57 | NA | *0.34±0.50 | 0.74±0.79 |
|  | Binary restenosis, % | | 18.90% | NA | 12.30% | 23.20% |

ACC/AHA: American College of Cardiology/American Heart Association; DES: drug-eluting stent(s); HD: hemodialysis; NA: not applicable; RA: rotational atherectomy; RPM: round per minute; SES: sirolimus-eluting stent(s);

**Table A-4**. Baseline Patient and Lesion Characteristics of the Included Orbital Atherectomy Studies

| **Study name** | | **ORBIT II** | **COAST** |
| --- | --- | --- | --- |
| **N** | | 443 | 100 |
| **Patient baseline  characteristics** | Age | 71.4 | 70.7 |
|  | Gender (% male) | 64.6% | 71% |
|  | Hypertension | 91.6% | 95% |
|  | Hyperlipidemia | 91.9% | 84% |
|  | Diabetes mellitus | 36.1% | 38% |
|  | Current smoker |  |  |
|  | Ever smoker | 66.1% | 73% |
|  | Family history of CAD |  |  |
|  | Chronic kidney disease |  | 24% |
|  | On hemodialysis | 0 | 13% |
| **Proportion of MACCEs and previous interventions** | Previous MI | 22.3% |  |
|  | Previous PCI |  |  |
|  | Previous CABG | 14.7% |  |
|  | Previous CVA | 8.8% | 11% |
|  | Previous PAD |  |  |
| **Lesion  characteristics** | Calcification level |  |  |
|  | ACC/AHA Classification B2/C |  |  |
|  | Chronic total occlusion |  |  |
|  | Bifurcation lesion |  |  |
|  | Minimum lumen diameter, pre (mm) |  | 0.49 |
|  | Minimum lumen diameter, post (mm) |  |  |
|  | Reference diameter (mm) | 3.1 |  |
|  | Mean Lesion length (mm) | 18.9 | 21.3 |

ACC/AHA: American College of Cardiology/American Heart Association; CABG: coronary artery bypass graft; CAD: coronary artery disease; CVA: cerebrovascular accident; MACCE: major adverse cardio-/cerebrovascular events; MI: myocardial infarction; PAD: peripheral artery disease; PCI: percutaneous coronary intervention

**Table A-5**. Weighted Average of Clinical Cohort Characteristics, Mean Lesion Length, and 12-month Target Lesion Revascularization Rate of Included Studies for Rotational and Orbital Atherectomy

|  | **Rotational Atherectomy** | **Orbital Atherectomy** |
| --- | --- | --- |
| N | 662 | 543 |
| Age | 72.8 | 71.3 |
| Male | 63.5% | 65.8% |
| Hypertension | 82.3% | 92.2% |
| Hyperlipidemia | 60.9% | 90.4% |
| Diabetes mellitus | 50.0% | 36.4% |
| Smoker | 56.7% | 67.4% |
| Previous MI | 21.7% | 22.2% |
| Previous CABG | 9.0% | 11.8% |
| Previous CVA | 12.0% | 9.2% |
| Chronic kidney disease | 31.0% | 24.0% |
| On hemodialysis | 13.9% | 2.4% |
| Mean lesion length (mm) | 28.7 | 19.3 |

CABG: coronary artery bypass graft; CVA: cerebrovascular accident; MI: myocardial infarction

**Table A-6**. Reported and Pooled Target Lesion Revascularization Rates

| Treatment | Study | N | | Proportion with Target Lesion  Revascularization at Month 12 | |
| --- | --- | --- | --- | --- | --- |
| **RA** |  | **662** |  | **15.7%** |  |
|  | Jinnouchi 2015 |  | 252 |  | 18.8% |
|  | Nishida 2013 (RA, non-HD) |  | 268 |  | 12.6% |
|  | Kyono 2011 (non-HD) |  | 63 |  | 9.7% |
|  | Tamekiyo 2009 (RA-SES) |  | 79 |  | 21.2% |
| **OA** |  | **543** |  | **5.0%** |  |
|  | ORBIT II |  | 443 |  | 4.7% |
|  | COAST |  | 100 |  | 6.3% |

HD: on hemodialysis; OA: orbital atherectomy; RA: rotational atherectomy; SES: sirolimus-eluting stent(s)

1. **Computation of remaining life years at age 74 (one year post-index procedure):**

Table A-2 below provides additional detail on the results of the claims data analysis discussed in the manuscript, specifically the number of underlying subjects contributing to the cost data in each category, as well as distributional information on the cost findings.

**Table A-7:** Results of claims data analysis. All amounts in Japanese Yen (JPY).

|  |  |  |  |  |
| --- | --- | --- | --- | --- |
|  |  |  |  |  |
| **Variable** | **Counts in dataset (N)** | **Mean Cost (JPY)** | **StdDev (JPY)** | **Median (JPY)** |
| Cost of index intervention, Rotablator | 1,373 | **2,504,198** | 1,684,446 |  |
| Cost of index intervention, other (PCI w/ and w/o stent) | 32,255 | 1,584,074 | 1,197,713 |  |
| Cost of 1st reintervention, all index procedure modalities | 7,288 | **1,184,220** | 606,929 | 1,050,806 |
| Cost of 1st reintervention, Rotablator index | 315 | 1,505,875 | 932,218 | 1,283,292 |
| Cost of 1st reintervention, PCI w/ no stent index | 968 | 1,233,871 | 865,793 | 1,031,034 |
| Cost of 1st reintervention, PCI w/ stent index | 6,005 | 1,159,310 | 523,953 | 1,043,424 |

1. **Computation of remaining life years at age 74 (one year post-index procedure):**

The health-economic study model computed the projected life year (LY) gain of patients treated with orbital atherectomy (OA) as opposed to rotational atherectomy (RA). To facilitate this computation, 12-month all-cause mortality for OA- and RA-treated patients reported in the included studies, respectively, were considered. The difference in the 12-month mortality rates of OA and RA was used to compute the total LY gain by multiplying the absolute difference in 12-month mortality by the remaining life years expected for the cohort at age 74 (index treatment of age 73 years plus 12-month follow-up).

Remaining life years were derived from the latest published Japanese lifetables and considered the gender-ratio of the study population. To account for the disease state of the study population, general population mortality was increased by applying a relative risk (RR) of 2.29. This factor was derived from a previous large-scale study reporting on survival in patients with severe artery calcification[5]. Specifically, this study reported age-adjusted mortality risks of 3.61 for patients with CAC score 11-100 (for the purposes of our analysis, this was assumed to be equivalent to the general population), and of 9.36 for patients with CAC score >1,000 (selected to reflect the current study’s population), resulting in a RR of 2.593.

As a result, remaining life years for our study cohort were estimated to be 7.72 years, as opposed to the 13.508 years reported in published Japanese lifetables. Sensitivity analyses evaluated the effect of variation in this parameter.

1. **Sensitivity analysis results (full detail)**

The tables below provide additional detail on the sensitivity analysis results discussed in the manuscript.

**Table A-8:** Additional One-way Sensitivity Analyses. All Amounts in Japanese Yen (JPY).


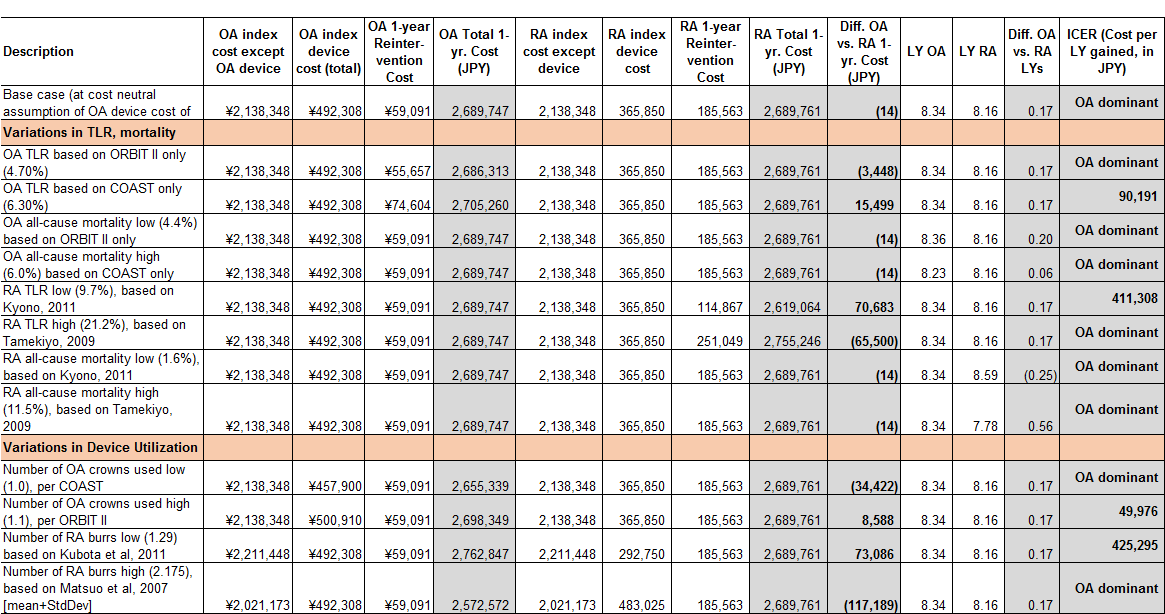


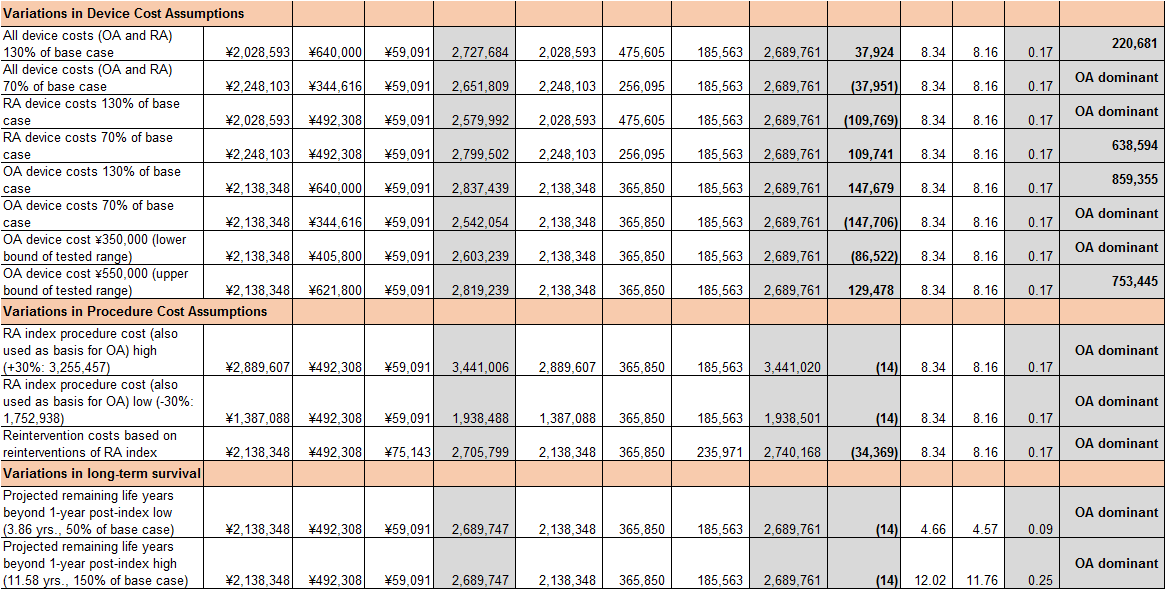


ICER: incremental cost-effectiveness ratio; JPY: Japanese Yen; LY: life years; OA: orbital rbital atherectomy; RA: rotational atherectomy; StdDev: standard deviation

**Table A-9:** Two-way Sensitivity Analysis. All Amounts in Japanese Yen (JPY).


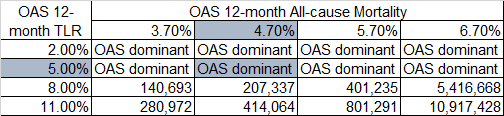


**References**

1. Tamekiyo H, Hayashi Y, Toyofuku M, Ueda H, Sakuma T, Okimoto T, et al. Clinical outcomes of sirolimus-eluting stenting after rotational atherectomy. Circ J. 2009;73:2042-9.

2. Jinnouchi H, Kuramitsu S, Shinozaki T, Kobayashi Y, Hiromasa T, Morinaga T, et al. Two-Year Clinical Outcomes of Newer-Generation Drug-Eluting Stent Implantation Following Rotational Atherectomy for Heavily Calcified Lesions. Circ J. 2015;79:1938-43.

3. Nishida K, Kimura T, Kawai K, Miyano I, Nakaoka Y, Yamamoto S, et al. Comparison of outcomes using the sirolimus-eluting stent in calcified versus non-calcified native coronary lesions in patients on- versus not on-chronic hemodialysis (from the j-Cypher registry). Am J Cardiol. 2013;112:647-55.

4. Kyono H, Kozuma K, Shiratori Y, Maeno Y, Iino R, Takada K, et al. Angiographic and clinical outcomes of 100 consecutive severe calcified lesions requiring rotational atherectomy prior to sirolimus-eluting stent implantation in hemodialysis and non-hemodialysis patients. Cardiovasc Interv Ther. 2011;26:98-103.

5. Budoff MJ, Shaw LJ, Liu ST, Weinstein SR, Mosler TP, Tseng PH, et al. Long-term prognosis associated with coronary calcification: observations from a registry of 25,253 patients. J Am Coll Cardiol. 2007;49:1860-70.
